# Genetic Polymorphisms of* IL17* and Chagas Disease in the South and Southeast of Brazil

**DOI:** 10.1155/2017/1017621

**Published:** 2017-04-02

**Authors:** Pâmela Guimarães Reis, Christiane Maria Ayo, Luiz Carlos de Mattos, Cinara de Cássia Brandão de Mattos, Karina Mayumi Sakita, Amarilis Giaretta de Moraes, Larissa Pires Muller, Julimary Suematsu Aquino, Luciana Conci Macedo, Priscila Saamara Mazini, Ana Maria Sell, Divina Seila de Oliveira Marques, Reinaldo Bulgarelli Bestetti, Jeane Eliete Laguila Visentainer

**Affiliations:** ^1^Post Graduation Program of Biosciences and Physiopathology, Department of Analysis Clinical and Biomedicine, Maringa State University, Maringa, PR, Brazil; ^2^Laboratory of Immunogenetics, Department of Molecular Biology, Medical School, São José do Rio Preto, SP, Brazil; ^3^Laboratory of Immunogenetics, Department of Basic Health Sciences, Maringa State University, Maringa, PR, Brazil; ^4^Department of Medical Clinic, Londrina State University, Londrina, PR, Brazil; ^5^Department of Cardiology and Cardiovascular Surgery, Medical School, São José do Rio Preto, SP, Brazil

## Abstract

The aim of this study was to investigate possible associations between genetic polymorphisms of* IL17A* G197A (rs2275913) and* IL17F* T7488C (rs763780) with Chagas Disease (CD) and/or the severity of left ventricular systolic dysfunction (LVSD) in patients with chronic Chagas cardiomyopathy (CCC). The study with 260 patients and 150 controls was conducted in the South and Southeast regions of Brazil. The genotyping was performed by PCR-RFLP. The A allele and A/A genotype of* IL17A* were significantly increased in patients and their subgroups (patients with CCC; patients with CCC and LVSD; and patients with CCC and severe LVSD) when compared to the control group. The analysis according to the gender showed that the A/A genotype of* IL17A* was more frequent in female with LVSD and mild to moderate LVSD and also in male patients with LVSD. The frequency of* IL17F* T/C genotype was higher in male patients with CCC and severe LVSD and in female with mild to moderate LVSD. The results suggest the possible involvement of the polymorphisms of* IL17A* and* IL17F* in the susceptibility to chronic Chagas disease and in development and progression of cardiomyopathy.

## 1. Introduction

Chagas disease (CD) is a serious anthropozoonosis common in the Americas and found mainly in endemic areas of the 21 Latin American countries [1]. On account of multinational initiatives, infection prevalence is progressively decreasing, and it is estimated that 6 to 8 million individuals are currently infected in the world, with an incidence of 28.000 cases a year [2]. Chagas disease presents an acute phase and a chronic phase. After the acute phase, most of the infected patients enter in the chronic phase of the disease and about 60 to 70% of infected persons are considered to have the indeterminate form (asymptomatic) of the disease [3–6]. After several years (10 to 30) of starting the chronic phase, 30 to 40% of the patients develop clinical manifestations known as the clinical forms: cardiac, digestive (mainly megaesophagus and megacolon), and cardiodigestive [5, 6]. The chronic Chagas cardiomyopathy (CCC) is the most severe form of the disease that affects 20 to 30% of the infected individuals. In endemic areas the disease is the main death cause in patients aged between 30 and 50 years [5, 7].

It is known that genetic variability and immunologic response influence the pathogenesis of the chronic phase of the disease. Associations were observed in several cytokine genes [8] with the susceptibility or protection against the development or progression of the CD and/or its clinical forms. The IL-17 is a proinflammatory cytokine secreted by T cells activated and expressed in different tissues. This cytokine takes part in inflammatory responses mediated by T cells and plays an important role in the tissue homeostasis and diseases progression [9]. The IL-17F presents a high degree of homology with the IL-17A (57% identical) [9] and seems to have a biological action similar to IL-17A, in vitro and in vivo, though significantly weaker [10]. The genes that codify them are mapped in the same chromosome, in the position 6p12 [9, 11].

Polymorphism in genes encoding cytokines may influence the level of cytokines production and, consequently, cause different immunological responses to different diseases. Previous studies show that genetic polymorphisms of* IL17A* G197A and* IL17F* T7488C affect the production of IL-17A and F, respectively [12, 13]. Such polymorphisms have already been associated with autoimmune and inflammatory diseases, as rheumatoid arthritis [14], periodontitis [15], and cancer, both gastric [16] and breast cancer [17]. To our knowledge, only one study involving the SNPs of* IL17A* and the CD [18] was found so far, and if we consider the SNPs of* IL17F* there are no related articles published yet. For this reason, our study aims to investigate whether the genetic polymorphisms of* IL17A* G197A (rs2275913) and* IL17F* T7488C (rs763780) were related to CD and/or the severity of the left ventricular systolic dysfunction (LVSD) in patients with CCC from North and Northeast regions of Parana and the Northeast region of São Paulo (states located in the South and Southeast of Brazil, resp.).

## 2. Material and Methods

### 2.1. Patients and Controls

For this study, 260 patients with chronic CD were selected from different municipalities in the North and Northwest regions of Parana and in the Northwest region of São Paulo. The patients were cared for in the Chagas Disease Laboratory in the State University of Maringa, the Clinical Hospital in Londrina, and the Base Hospital of the Medical School in São José do Rio Preto. All patients were submitted to a resting electrocardiogram (ECG) exam and a two-dimensional echocardiography. Patients who presented a normal ECG were classified as patients without CCC and patients with electrocardiographic changes, common to CCC, were classified as patients with CCC. The severity of the LVSD was measured according to the left ventricular ejection fraction (LVEF) and the Teichhoolz method was applied following the II Brazilian Guideline for Severe Heart Diseases [19]. Patients with CCC were classified considering the (LVEF) in three different groups: patients without LVSD (LVEF > 60%); patients with mild to moderate LVSD (LVEF 40–60%); and patients with severe LVSD (LVEF < 40%). To all statistical analysis were considered the following groups: all Chagas disease patients (CD), chronic Chagas cardiomyopathy patients (CCC), without Chagas cardiomyopathy patients (without CCC), chronic Chagas cardiomyopathy patients with LVSD (with LVSD), chronic Chagas cardiomyopathy patients without LVSD (without LVSD), patients with mild to moderate LVSD (Mild/moderate LVSD), and patients with severe LVSD (severe LVSD).

The control group was composed of 150 individuals, healthy and nonrelated, patient's spouses, and contacts retirement communities' residents with negative serology to* T. cruzi* antigens. The clinicopathological features of patients and controls are presented in Table 1. No significant differences were observed among groups in terms of gender, but differences in age were observed between CCC and without CCC patients (63.9 ± 10.2 versus 58.6 ± 7.8, respectively; *P* ≤ 0.05). Due to the significant miscegenation of Brazilian population we consider patients and controls as a mixed ethnic group (Caucasians, Mulattos, and Blacks) according to Parra et al. (2003) [20]. Mean age, gender rates, and residence in the same geographical areas were carefully matching to select the groups.

The laboratory diagnosis of CD in patients and controls was made by ELISA (Enzyme-Linked ImmunoSorbent Assay) test, in serum or plasma, using the immunoassay “Chagas” from Abbott Laboratories (Santiago, Chile). In cases of weak reagent, the diagnosis was confirmed by the indirect immunofluorescence test (IIFT) with the IMUNOCRUZI® antigen (Biolab, Rio de Janeiro, Brazil) or ELISAcruzi (bioMerieus SA, Brazil), respecting the manufacturer's instructions.

The Ethics committees from each institution have approved this study, as seen in the protocols they have registered (012/2010-COPEP-UEM, CAAE 0296.0.093.000–09; FAMERP - # 009/2011), and written informed consent was obtained from all subjects prior to participation.

### 2.2. DNA Extraction and Genotyping

The extraction method used in this research was the salting-out adapted [21]. The genomic DNA was extracted from 250 *μ*L of buffy-coat obtained from 5 mL of peripheral blood collected in tubes with EDTA (Ethylenediaminetetraacetic acid). The material's concentration and purity were determined by NanoDrop 2000® equipment (Thermo Scientific, Wilmington, USA).

The SNPs in* IL-17A* (rs2275913) and* IL-17F* (rs763780) were genotyped using PCR-RFLP (Polymerase Chain Reaction-Restriction Fragment Length Polymorphism) [15]. The primers sequences to* IL17A* G197A were sense 5′-AACAAGTAAGAATGAAAAGAGGACATGGT-3′ and antisense 5′-CCCCCAATGAGGTCATAGAAGAATC-3, while to* IL17F* T7488C they were sense 5′-ACCAAGGCTGCTCTGTTTCT-3′ and antisense 5′-GGTAAGGAGTGGCATTTCTA-3′. The reaction of DNA amplification was made in a total volume of 30 *μ*L, containing 100 ng of genomic DNA, 1,0 *μ*M from each primer, 200 *μ*M from each dNTP, 2,0 mM of MgCl2, 3 *μ*L of 10x PCR buffer, and 1,5 U of Taq DNA polymerase (Invitrogen Life Technologies, Grand Island, NY, USA). The PCR products were digested during one hour submitted to 37°C with the enzyme* Xag*I (Fermentas, Canada) to* IL17A* G197A and the enzyme* Nla*III (New England Biolabs) to* IL17F* T7488C and, subsequently, separated by agarose gel electrophoresis to 3,5% with SYBR Green (Invitrogen Life Technologies, Grand Island, NY, USA).

### 2.3. Statistical Analysis

The allele and genotype frequencies of* IL17A* G197A and* IL17F* T7488C were estimated and the genotype distribution was evaluated to Hardy-Weinberg balance [22]. The association tests were realized to the codominant, dominant, recessive, overdominant, and log-additive genetic inheritance models. The *P* ≤ 0.05 values were considered statistically significant to Chi-square test with Yates correction and logistic regression. The statistical comparisons between these groups were realized and the estimated risk to develop CD and/or CCC in individuals who hold genetic polymorphisms was calculated by determination of OD (Odds Ratio) with 95% of confidence interval, adjusted by gender and age. All statistical analysis was performed using the software SNPStats (http://bioinfo.iconcologia.net/index.php) [23] and the OpenEpi program, version 3.03a (http://www.openepi.com/Menu/OE_Menu.htm).

## 3. Results

The ratio distributions of genotype frequency for all analyzed genes were in Hardy-Weinberg equilibrium (*P* > 0.05). In order to evaluate the possible association of* IL17A* G197A and* IL17F* T7488C SNPs and Chagas disease, the allele and genotype frequencies between patients (CD) and their subgroups (CCC, without CCC, with LVSD, without LVSD, Mild/moderate LVSD, severe LVSD) and controls were compared (Table 2). Statistically significant differences were observed for A allele and A/A genotype of* IL17A* but no significant difference was found to* IL17F*.

The A allele frequency of* IL17A* was significantly higher in the CD patients when compared to controls (*P* = 0.032, OR = 1.46, 95% CI = 1.05–2.05). The same was found when CCC patients (*P* = 0.021, OR = 1.52, 95% CI = 1.08–2.15), patients with CCC and LVSD (*P* = 0.009, OR = 1.73, 95% CI = 1.15–2.59), and patients with CCC and severe LVSD (*P* = 0.009, OR = 1.97, CI = 1.20–3.21) were compared to controls.

The A/A genotype was more frequent in the CD patients than in the control group and statistically significant differences were observed in more than one model of genetic inheritance (Codominant: *P* = 0.019, OR = 4.53, 95% CI = 1.31–15.73; Recessive: *P* = 0.0089, OR = 4.12, 95% CI = 1.20–14.13; Log-additive: *P* = 0.02, OR = 1.50, 95% CI = 1.06–2.13). Same results can be seen when the subsets are compared: CCC versus controls (Codominant: *P* = 0.01, OR = 5.16, 95% CI = 1.47–18.14; Recessive: *P* = 0.0048, OR = 4.67, 95% CI = 1.35–16.18; Log-additive: *P* = 0.013, OR = 1.57, 95% CI = 1.09–2.24); patients with CCC and LVSD versus controls (Codominant: *P* = 0.006, OR = 6.81, 95% CI = 1.77–26.29; Dominant: *P* = 0.034, OR = 1.73, 95% CI = 1.04–2.87; Recessive: *P* = 0.0045, OR = 5.73, 95% CI = 1.52–21.64; Log-additive: *P* = 0.0046, OR = 1.85, 95% CI = 1.20–2.85); patients with CCC and severe LVSD versus controls (Codominant: *P* = 0.0047, OR = 9.64, 95% CI = 2.28–40.85; Recessive: *P* = 0.002, OR = 8.18, 95% CI = 2.00–33.51; Log-additive: *P* = 0.057, OR = 2.11, 95% CI = 1.24–3.60). For all comparisons, the recessive inherence model was the best according Akaike information criteria (AIC). It means that two copies of A are necessary to change the risk, so G/A or G/G have the same effect. No difference was observed when allele and genotype frequencies of* IL17A* were compared between patients with CCC and patients without CCC. Likewise, no association was observed when the progression of cardiac forms was considered: the different forms (without LVSD, with LVSD, mild/moderate LVSD, and severe LVSD) were compared with each other and no statistically significant difference was noticed.

After stratifying according to gender significant differences were observed for* IL17A* and* IL17F* genotype frequencies when the progression of cardiac form was evaluated. The* IL17A* A/A genotype was more frequent in female with LVSD (OR = 6.63, 95% CI = 1.21–36.40) and with mild/moderate LVSD (OR = 7.57, 95% CI = 1.07–53.40) than in the control group, although not significant males with LVSD also had higher frequency of AA genotype compared to controls (13.5 versus 7.84%, resp.) (Table 3). In relation to* IL17F*, the T/C genotype was more frequent in male patients with severe LVSD when compared to other groups: without LVSD (OR = 4.82, 95% CI = 1.55–14.98), with mild/moderate LVSD (OR = 6.00, 95% CI = 1.18–30.63), without CCC patients (OR = 6.70, 95% CI = 1.19–37.53), and controls (OR = 3.40, 95% CI = 1.24–9.31). In female statistical difference was not observed, although T/C was higher in mild/moderate LVSD (17%) when compared to others patients and control (Table 4). Considering the variable age, no significant difference was observed between* IL17* SNPs and CD and/or the severity of the left ventricular systolic dysfunction (LVSD).

## 4. Discussion

The identification of genes that are candidates for susceptibility or protection against CD has major implications, not only to better understand the pathogenesis of the disease, but also to control and develop therapeutic strategies. In this study, a possible association between the genetic polymorphisms of* IL17A* G197A and* IL17F* T7488C with CD and the severity of CCC was investigated in a population from South and Southwest regions in Brazil.

In this study, the* IL17A* A allele and the A/A genotype were more frequent in CD and CCC patients, female with LVSD or mild/moderate LVSD and male with LVSD when compared to control. The risk to severe LVSD was observed in male carrying the* IL17F* T/C genotype. The* IL17* polymorphism could be correlated to the risk of disease, indicating susceptibility to chronic Chagas disease and increasing risk of severe cardiomyopathy when gender was considered in multivariate analyses. The mutant allele A of* IL17A* was associated with a higher production of IL-17 [12] and the IL-17F activity is similar to IL-17A, although significantly weaker [10, 12, 13]. Based on these findings, it is possible to infer that the higher production of IL-17, a proinflammatory cytokine, could contribute to tissue damage and might be related to the development and progression of CCC in this population.

Considering the IL-17 biological function in Chagas disease, Guedes et al. [24] showed that the neutralization of IL-17 in mice BALB/c infected with* T. cruzi* has resulted in a higher recruitment of inflammatory cells to the cardiac tissue in the acute phase of the infection, leading to an increase in myocarditis and, consequently, premature death, despite the reduction of the local parasitism. Miyazaki et al. [25] have reported the importance of the IL-17 in the* T. cruzi* infection and the cardiac inflammation control in CD. They observed that in the experimental acute infection with* T. cruzi*, disabled mice in IL-17 presented a higher mortality rate and parasitemia when compared to the group control (C57BL/6, wild type), as well as a lower expression of cytokines, as IFN-*γ*, IL-6, and TNF-*α*, suggesting a protective role of IL-17 in the acute phase of the disease. The neutralization of IL-17 also resulted in a higher production of IL-12, IFN-*γ*, TNF-*α*, chemokines, and its receptors, indicating that the IL-17 may perform a role in the control of cardiac inflammation, through the modulation of Th1 response. On the other hand, Magalhães et al. [26] showed that in Chagas patients with cardiac form the total lymphocytes and the Th17 cells presented a low expression of IL-17A in comparison to the patients with the indeterminate form and control group, and the analysis of correlation between IL-17A and the cardiac function showed that the high expression of this cytokine was associated with a better clinical outcome in the human CD, according to values of the ejection fraction and left ventricular diastolic diameter, indicating a protective role against the severity of CCC.

Five SNPs of* IL17A* were analyzed in patients with CD in a population of an endemic region of Colombia. >The SNP rs8193036 was associated with the protection against* T. cruzi* infection and the development of CCC. Meanwhile for the SNP rs2275913, the same SNP evaluated in this study, the frequency of allele A was higher in patients than in controls and significant difference was observed, although significance was lost after the correction [18]. We observed that* IL17A* A allele and AA genotype were higher in Chagas disease as well in CCC with or without LVSD, but no difference was observed between CD or CCC patients. However, after stratifying according to gender, female with* IL17A* AA genotype had risk of developing mild/moderate LVSD (approximately seven), as male to develop LVSD (although not significant); and male with* IL17F* T/C genotype had higher risk to develop severe LVSD compared to other cardiac form and controls.

A study conducted by Peng et al. [27] in Chinese patients with dilated cardiomyopathy did not find association with* IL17A* G197A and* IL17F* T7488C polymorphism. However, after stratification by gender, the* IL17F* was associated with dilated cardiomyopathy in male patients that present the T/C-C/C genotypes, suggesting that the presence of the rare allele (C) might be associated with the disease in these patients. In this study we found that the* IL17F* T/C genotype was associated with developing severe LVSD in male patients when the sample stratification by gender was done.

The risk of development severe cardiac form in male with CD was showed in two Brazilian studies. Rassi et al. [28] showed that gender (male) and left ventricular systolic dysfunction on echocardiography are potential risk factors for death in subjects with CD. They evaluated a cohort of 424 Brazilian outpatients followed for about eight years and confirmed the results in 153 patients of other Brazilian community hospital. Faé et al. [29] observed a higher risk of developing severe forms of cardiomyopathy in men (OR = 8.75), corroborating the results of this study.

The present study has potential limitations. The major limitation was the number of patients limiting the significance of results and consequently no strong association could be found, principally when independent multiple comparisons were carried out. However, the risk of population stratification bias, due to differences in ethnic background, was minimized by matching patients with controls individuals of the same ethnic background. Mean age, gender rates, and residence in the same geographical areas were carefully matching to select the groups. Another limitation was that* IL17* gene expression or serum levels were not evaluated.

## 5. Conclusions

In these South and Southeast Brazilian patients, the* IL17A* polymorphisms, AA genotype and A allele, were associated with susceptibility to chronic CD and the severity of the left ventricular systolic dysfunction (LVSD). In addition, the* IL17A* A/A genotype was associated with mild/moderate LVSD in female patients, whereas the* IL17F* T/C genotype was associated with severe LVSD in male patients. These results suggest the possible involvement of the polymorphisms of* IL17A* and* IL17F* in the susceptibility to chronic CD and in development and progression of CCC. Additional studies are needed to confirm these results and for understanding the functional role polymorphism in CD.

## Figures and Tables

**Table 1:** Characteristics of the chronic Chagas disease patients and controls from South and Southeast of Brazil.

	CD patients	CCC	Without CCC	Control
	*N* = 260	*N* = 212	*N* = 48	*N* = 150
Gender^**a**^ *n* (%)				
Male	121 (46.5)	97 (45.8)	24 (50.0)	74 (49.3)
Female	139 (53.5)	115 (54.2)	24 (50.0)	76 (50.7)
Age^**b**^				
Min-max	31–90	31–90	38–76	28–100
Mean ± SD (year)	62.9 ± 10.0	63.9 ± 10.2	58.6 ± 7.8	62.3 ± 17.4

CCC, patients with chronic Chagas cardiomyopathy; Min, minimum age; Max, maximum age; SD, standard deviation.

^**a**^No statistically significant difference was observed between the groups for gender.

^**b**^Statistically significant difference was observed between the groups for age: CCC versus without CCC.

**Table 2:** Genotypes and allele frequencies distribution of* IL17A* rs2275913 and *IL17F* rs763780 in Chagas disease patients and controls in a population from South and Southeast of Brazil.

Allele/genotype *n* (%)	CD patients	CCC	Without LVSD	With LVSD	Mild/moderate LVSD	Severe LVSD	Without CCC	Control
	*N* = 260	*N* = 212	*N* = 109	*N* = 103	*N* = 52	*N* = 51	*N* = 48	*N* = 150
*IL17A* G197A								
G	369 (71.2)	297 (70.4)	159 (72.9)	138 (67.6)	72 (70.6)	66 (64.7)	72 (75.0)	235 (78.3)
A	**149 (28.8)** ^**a**^	**125 (29.6)** ^**b**^	59 (27.1)	**66 (32.4)** ^**c**^	30 (29.4)	**36 (35.3)** ^**d**^	24 (25.0)	**65 (21.7)**
GG	130 (50.2)	104 (49.3)	58 (53.2)	46 (45.1)	24 (47.0)	22 (43.1)	26 (54.2)	88 (58.7)
GA	109 (42.1)	89 (42.2)	43 (39.5)	46 (45.1)	24 (47.0)	22 (43.1)	20 (41.7)	59 (39.3)
AA	**20 (7.7)** ^**e**^	**18 (8.5)** ^**f**^	8 (7.3)	**10 (9.8)** ^**g**^	3 (6.0)	**7 (13.8)** ^**h**^	2 (4.1)	**3 (2.0)**

*IL17F* T7488C								
T	484 (93.1)	394 (92.9)	207 (94.9)	187 (90.8)	97 (93.3)	90 (88.2)	90 (93.7)	282 (94.0)
C	36 (6.9)	30 (7.1)	11 (5.1)	19 (9.2)	7 (6.7)	12 (11.8)	6 (6.3)	18 (6.0)
TT	224 (86.2)	182 (85.8)	98 (89.9)	84 (81.6)	45 (86.5)	39 (76.5)	42 (87.5)	132 (88.0)
TC	36 (13.8)	30 (14.2)	11 (10.1)	19 (18.4)	7 (13.5)	12 (23.5)	6 (12.5)	18 (12.0)

CCC: patients with chronic Chagas cardiomyopathy; LVSD: left ventricular systolic dysfunction; Recessive model: AA versus GA + GG; OR: *odds ratio*; CI: confidence interval. Adjustment of the genotypic differences for the effect of age and gender was applied.

^**a**^ *P* = 0.032. OR = 1.46 and 95% CI = 1.05–2.05; CD patients versus controls.

^**b**^ *P* = 0.021. OR = 1.52 and 95% CI = 1.08–2.15; CCC versus controls.

^**c**^ *P* = 0.009. OR = 1.73 and 95% CI = 1.15–2.59.With LVSD versus controls.

^**d**^ *P* = 0.009. OR = 1.97 and 95% CI = 1.20–3.21. Severe LVSD versus controls.

^**e**^Recessive model: *P* = 0.009; OR = 4.12; 95% CI = 1.20–14.13. CD patients versus controls.

^**f**^Recessive model: *P* = 0.005; OR = 4.67; 95% CI = 1.35–16.18. CCC versus controls.

^**g**^Recessive model: *P* = 0.005; OR = 5.73; 95% CI = 1.52–21.64. With LVSD versus controls.

^**h**^Recessive model: *P* = 0.002; OR = 8.18; 95% CI = 2.00–33.51. Severe LVSD versus controls.

**Table 3:** Genotype frequencies of *IL17A *rs2275913 in Brazilian patients with LVSD in chronic Chagas cardiomyopathy, stratified according to gender.

Gender	*IL17A* G197A	With LVSD *n* (%)	Mild/moderate LVSD *n* (%)	Control *n* (%)
Male	GG	23 (45.1)	13 (59.1)	43 (58.11)
	GA	24 (47.06)	9 (40.9)	30 (40.54)
	AA	4 (7.84)	0	1 (1.35)

Female	GG	23 (45.1)	11 (37.93)	45 (59.21)
	GA	22 (43.14)	15 (51.72)	29 (38.16)
	AA	**6 (11.76)** ^**a**^	**3 (10.35)** ^**b**^	**2 (2.63)**

LVSD, chronic Chagas cardiomyopathy patients with left ventricular systolic dysfunction; OR, *odds ratio*; CI, confidence interval.

Data adjusted by age.

Only significant results are showed.

^**a**^OR = 6.63 and 95% CI = 1.21–36.40; with LVSD versus control.

^**b**^OR = 7.57 and 95% CI = 1.07–53.40; mild/moderate LVSD versus control.

**Table 4:** Genotype frequencies of *IL17F *rs753780 in Brazilian Chagas disease patients with chronic cardiomyopathy, stratified according to gender.

Gender	*IL17F* T7488C	Without LVSD *n* (%)	Mild/moderate LVSD *n* (%)	Severe LVSD *n* (%)	Without CCC *n* (%)	Controls *n* (%)
Male	TT	40 (86.96)	21 (91.3)	19 (65.5)	22 (45.8)	64 (86.49)
	TC	**6 (13.04)** ^**a**^	**2 (8.7)** ^**b**^	**10 (34.5)**	**2 (4.2)** ^**c**^	**10 (13.51)** ^**d**^

Female	TT	58 (92.06)	24 (82.8)	20 (90.9)	20 (41.7)	68 (89.47)
	TC	5 (7.94)	5 (17.2)	2 (9.1)	4 (8.3)	8 (10.53)

CCC, chronic Chagas cardiomyopathy; LVSD, left ventricular systolic dysfunction; OR, odds ratio; CI, confidence interval.

Data adjusted by age.

Only significant results are showed.

^**a**^OR = 4.82 and 95% CI = 1.55–14.98; severe LVSD versus without LVSD.

^**b**^OR = 6.02 and 95% CI = 1.18–30.78; severe LVSD versus mild/moderate LVSD.

^**c**^OR = 6.70 and 95% CI = 1.19–37.53; severe LVSD versus without CCC patients.

^**d**^OR = 3.40 and 95% CI = 1.24–9.31; severe LVSD versus controls.
